# Supplementary material for: High-Capacity Free-Space Optical Communications Between a Ground Transmitter and a Ground Receiver via a UAV Using Multiplexing of Multiple Orbital-Angular-Momentum Beams
Source: Sci Rep. 2017 Dec 12;7:17427. doi: 10.1038/s41598-017-17580-y (PMC5727312; doi:10.1038/s41598-017-17580-y)
Supplement: Supplementary file 1 — Supplementary material [file 41598_2017_17580_MOESM1_ESM.pdf]

High-Capacity Free-Space Optical Communications Between a Ground Transmitter and a Ground Receiver via a UAV Using Multiplexing of Multiple Orbital-Angular-Momentum Beams

Long Li[1,*,+], Runzhou Zhang[1,+], Zhe Zhao[1], Guodong Xie[1], Peicheng Liao[1], Kai Pang[1],

Haoqian Song[1], Cong Liu[1], Yongxiong Ren[1], Guillaume Labroille[2], Pu Jian[2],

Dmitry Starodubov[1], Brittany Lynn[3], Robert Bock[4], Moshe Tur[5], Alan E. Willner[1,*]

[1]Department of Electrical Engineering, U. of Southern California, Los Angeles, CA 90089, USA

[2]CAILabs Labs, Rennes 35200, France

[3]Space & Naval Warfare Systems Center, Pacific, San Diego, CA 92152, USA

[4]R-DEX Systems, Marietta, GA 30068, USA

[5]School of Electrical Engineering, Tel Aviv University, Ramat Aviv 69978, Israel

[+]These authors contributed equally to this work

Corresponding email: longl@usc.edu, willner@usc.edu

1. Measurements of OAM beam quality reflected from the retro-reflector:

We measure the effects of retro-reflector on the OAM beams quality. The experimental setup is shown in Fig. S1a. An OAM beam at 1550 nm is generated by launching a collimated Gaussian beam with a beam waist of 2.2 mm onto a spatial light modulator (SLM) loaded with a specific pattern. The generated OAM beam is then reflected by a retro-reflector with a diameter of 2 inches. We measure the OAM power spectrum of the transmitted OAM beam (shown in Fig. S1b) and the reflected OAM beam (shown in Fig. S1c) when the transmitted OAM beam is launched onto different positions of the retro-reflector (shown in Fig. S1d). We observe that the OAM order of the reflected beam is the negative of that of the incoming beam, but its OAM power spectrum

shows similar power coupled into the desired mode as well as crosstalk to the neighbouring modes.

2. Specifications of the UAV and the gimbal:

The retro-reflector is mounted on a gimbal and carried by an octocopter UAV. The gimbal targets the retro-reflector to the ground platform using GPS information with an accuracy of ~2-m, i.e., ~12° at a distance of 10 m, and 1.2° at a distance of 100 m. The angular range of the incoming beam that our retro-reflector could reflect is between ~±15° referring to its axis. Therefore this targeting accuracy would satisfy the requirements of our tracking system. The gross weight of the retro-reflector is 4 kg, which is within the maximum payload of the gimbal (5.5 kg). The gross weight of the gimbal is 1.5 kg, thus a total weight of 5.5 kg is carried by the UAV, which is also within its maximum payload of 9 kg.

3. Method for optical signal generation and coherent detection

The schematic diagram of the optical modules is shown in Fig. S2. A 1550-nm light is generated by a laser source and modulated by a Lithium Niobate ($LiNbO_3$) In-phase/Quadrature (I/Q) modulator. A polarisation controller (PC) is used to control the polarisation of the input light to match with that of the I/Q modulator. A 20-GHz clock source is used to generate the 20-Gbaud (i.e., 40-Gbit/s) QPSK signal with a pseudorandom binary sequence (PRBS) of 2^{31}-1. After being amplified by the EDFA, the modulated light is sent to the on field OAM-multiplexed FSO communications link. For coherent optical detection, the received signal is amplified by an EDFA, and the optical signal detector (homodyne receiver) is used to detect the received signal, and calculate its BER.

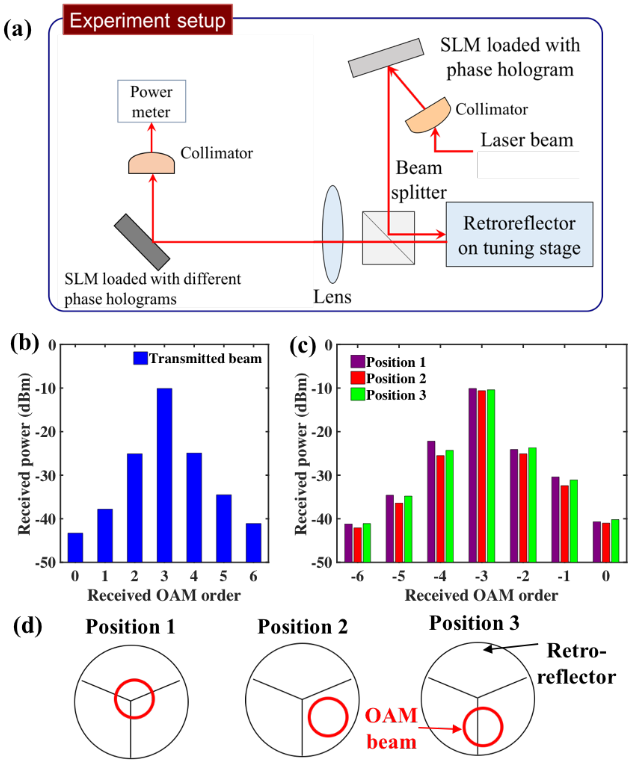

Figure S1 | Measurements on the effect of retro-reflector on the OAM beams. (**a**) Experimental setup. (**b**) Measured OAM power spectrum of the transmitted OAM +3 beam. (**c**) Measured OAM power spectrums of the reflected OAM beam when OAM +3 beam is launched on different positions of the retro-reflector. (**d**) Illustration of the different positions on which the OAM +3 beam is launched. The retro-reflector has a diameter of 2 inches. After reflection, a beam's OAM order changes between + ℓ and - ℓ.

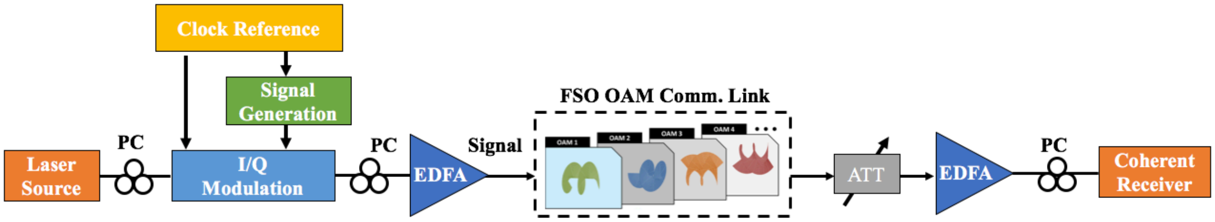

Figure S2 | Schematic diagram of signal generation and coherent optical detection. ATT: optical attenuator; Comm.: communications; EDFA: Erbium-doped fibre amplifier; FSO: free-space optical; I/Q: in-phase and quadrature; OAM: orbital angular momentum; PC: polarisation controller.
